# Effective public health measures to mitigate the spread of COVID-19: a systematic review

**DOI:** 10.1186/s12889-021-11111-1

**Published:** 2021-05-29

**Authors:** Imen Ayouni, Jihen Maatoug, Wafa Dhouib, Nawel Zammit, Sihem Ben Fredj, Rim Ghammam, Hassen Ghannem

**Affiliations:** Department of Epidemiology, University Hospital Farhat Hached Sousse, Faculty of Medicine of Sousse, Sousse, Tunisia

**Keywords:** Public heath interventions, Non-pharmaceutical measures, Prevention, COVID-19, Systematic review

## Abstract

**Background:**

In December 2019, a novel coronavirus (2019-nCoV) was recognized in Wuhan, China. It was characterised by rapid spread causing a pandemic. Multiple public health interventions have been implemented worldwide to decrease the transmission of the 2019 novel coronavirus disease (COVID-19). The objective of this systematic review is to evaluate the implemented public health interventions to control the spread of the outbreak of COVID-19. **Methods:** We systematically searched PubMed, Science Direct and MedRxiv for relevant articles published in English up to March 16, 2021. We included quasi experimental studies, clinical trials, cohort studies, longitudinal studies, case-control studies and interrupted time series. We included the studies that investigated the effect of the implemented public health measures to prevent and control the outbreak of 2019 novel coronavirus disease (COVID-19).

**Results:**

The database search using the predefined combinations of Mesh terms found 13,497 studies of which 3595 in PubMed, 7393 in Science Direct 2509 preprints in MedRxiv. After removal of the duplicates and the critical reading only 18 articles were included in this systematic review and processed for data extraction.

**Conclusions:**

Public health interventions and non-pharmaceutical measurements were effective in decreasing the transmission of COVID-19. The included studies showed that travel restrictions, borders measures, quarantine of travellers arriving from affected countries, city lockdown, restrictions of mass gathering, isolation and quarantine of confirmed cases and close contacts, social distancing measures, compulsory mask wearing, contact tracing and testing, school closures and personal protective equipment use among health workers were effective in mitigating the spread of COVID-19.

## Introduction

In the twenty-first century, two highly pathogenic human coronaviruses (HCoVs) severe acute respiratory syndrome coronavirus (SARS-CoV) and Middle East respiratory syndrome coronavirus (MERS-CoV) emerged from animal reservoirs to cause global epidemics. In December 2019, yet another pathogenic HCoV, 2019 novel coronavirus (2019-nCoV), was recognized in Wuhan, China, and has caused serious illness and death [1]. This novel coronavirus is characterised by rapid spread and high contagiousness [2] which caused a pandemic as it was spreading rapidly between and within the countries. As of 18 March 2021 severe acute respiratory syndrome coronavirus 2 (SARS-CoV-2) has caused more than 121.8 million cases and 2.69 deaths [3] affecting 221 countries and territories. Since the beginning of the COVID-19 pandemic several public health interventions have been implemented worldwide to reduce the transmission of the SARS-CoV-2. In previous experiences, like the 1918–19 H1N1 influenza pandemic where no treatments or vaccines were available to treat or prevent the disease multiple non-pharmaceutical interventions were successful at reducing case numbers and have shown to be effective when implemented early in the epidemic. Those interventions include travel bans and restrictions, schools and workplace closures, isolating infected persons, quarantine of exposed persons, social distancing and cancellation of mass gathering events. Those interventions have shown to be effective ways to respond to the outbreak when implemented early in the epidemic [4–9]. However the effectiveness of those interventions whether applied alone or simultaneously still unclear and results from previous modelling studies are inconsistent [10].

Within this systematic review we aimed to evaluate the public health interventions and the non-pharmaceutical control measures that have been implemented worldwide to mitigate and control the spread of the outbreak of 2019 novel coronavirus disease (COVID-19).

## Methods

### Search strategy and selection criteria

We conducted a systematic review in accordance with the Preferred Reporting Items for Systematic Reviews and Meta-Analyses (PRISMA) guidelines [11]. The protocol of this systematic review was published on PROSPERO (registration number CRD42020196018). Given the nature of this research study no approval by an institutional review board was necessary. We systematically searched PubMed, Science Direct and MedRxiv for relevant articles published in English up to March 16, 2021 using the following combinations of terms in PubMed: (((“public health”[MeSH Terms]) OR “preventive medicine”[MeSH Terms])) AND “covid 19”[MeSH Terms]; (health knowledge, attitudes, practice [MeSH Terms]) AND covid 19[MeSH Terms]; (((“covid 19”[MeSH Terms]) AND (“epidemiology”[MeSH Terms])) OR (public health interventions [Title/Abstract])) OR (epidemiological assessment [Title/Abstract]); (((“covid 19”[MeSH Terms]) AND (“social distancing”[Title/Abstract])) OR (“quarantine”[MeSH Terms])) OR (“isolation”[Title/Abstract]); (“covid 19”[MeSH Terms]) AND (“contact tracing”[MeSH Terms]); (“covid 19”[MeSH Terms]) AND (“lockdown”[Title/Abstract]). In Science Direct and MedRxiv we used the following terms: “Public Health measures” and “covid-19”.

### Study eligibility and quality assessment

We included articles published only in English language up to March 16, 2021, clinical trials, quasi experimental studies, cohort studies, longitudinal studies, case-control studies, and interrupted time series. The studies that investigated the effect of the non-pharmaceutical interventions such as social distancing, lockdown, quarantine, mobility and travel restrictions, border control measures, contact tracing, isolation of cases that have been implemented to mitigate, prevent and control the outbreak of 2019 novel coronavirus disease (COVID-19). We excluded articles published in a language other than English; narrative literature reviews, policy reviews, case studies, case reports, case series, cross-sectional studies, ecological studies, commentaries, editorials, letters, point of views, simulation studies, modelling studies, prediction studies, qualitative studies,systematic reviews and meta-analysis.

The database search was conducted by one author (AI) who did the tiles and abstracts screening in order to identify the eligible studies for full text review with referral to (MJ) and (DW). Both authors (AI, DW) did the full text review of the studies that potentially met eligibility criteria and checked their relevance with referral to a third author (MJ) in case of discordance. Any discrepancy between the reviewers was resolved by discussion.

### Data analysis

Two authors (AI, DW) did the data extraction using a standardized form to collect the relevant data from each article. The form included study identification features (authors, article title, country of origin), study characteristics (aim of the study, study design), characteristics of the studied population, public health interventions that has been implemented (description of the intervention(s) and control(s) if applicable), outcomes and authors’ conclusions. The included studies were evaluated for quality and risk of bias using the Effective Public Health Practice Project (EPHPP) quality assessment tool quantitative studies [12]. All studies were independently assessed for quality by two reviewers (AI, DW), with disagreements resolved by discussion until full consensus was reached with referral to (MJ) and (ZN). Level of evidence and grade of recommendation of the included studies were assessed according to the Scottish Intercollegiate Guidelines Network (SIGN) system [13].

## Results

**Fig. 1:**
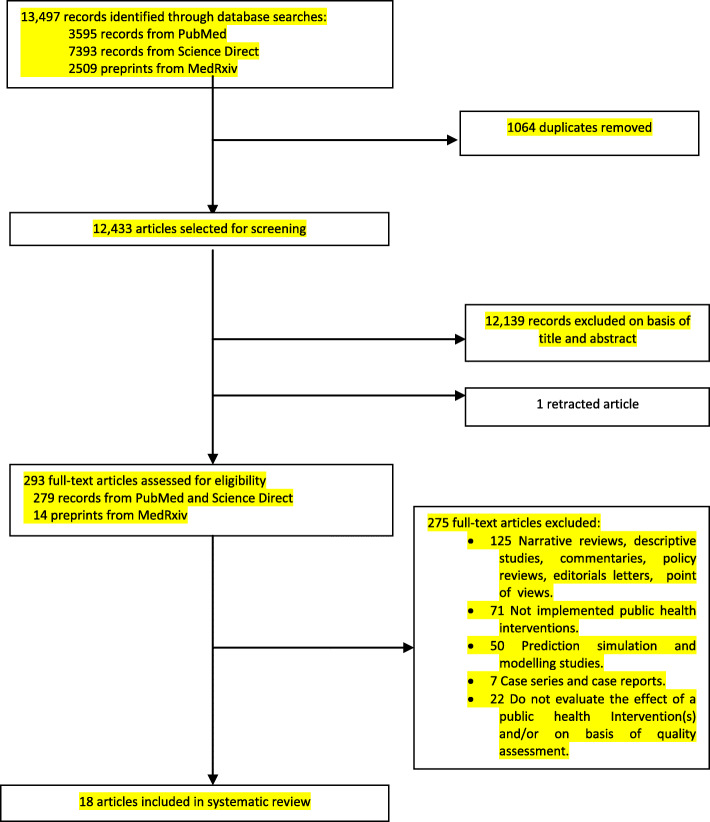
Study selection

The database search in PubMed and Science Direct using the predefined combinations of Mesh terms found 13,497 studies of which 3595 in PubMed, 7393 in Science Direct 2509 preprints in MedRxiv. After removal of the duplicates 12,433 articles remained. During the screening stage one article was excluded as it was retracted and 12,139 records were excluded on basis of title and abstract. After the critical reading of the 293 remaining articles, 275 articles were excluded seeing that they didn’t meet the eligibility criteria and only 18 articles were included in this systematic review and processed for data extraction. Fig. 1 summarized the described outcomes. The characteristics of the included studies and the main results were summarized Table 1 including the following items: authors, country, study design, objective, methods and main outcomes. For the quality assessment results the quality of 14 (77.77%) included studies [14–16, 19–21, 23–29, 31] was moderate, the quality of two studies [18, 22] was strong and the quality was weak for the two remaining studies [17, 30] (Table 2). As for the results of the level of evidence and grade of recommendation assessment, six studies had low level of evidence and low grade of recommendation [16, 17, 19, 21, 24, 30]. Ten studies had moderate level of evidence and low grade of recommendation [14, 15, 20, 22, 23, 25–29], one study had moderate level of evidence and moderate grade of recommendation [18] and only one study had high level of evidence and high grade of recommendation [31] (Table 3). Three studies [16, 22, 26] have found that travel entry restrictions and bans, borders control measures and quarantine of travellers especially the ones arriving from affected countries along with other interventions was effective in reducing the spread of COVID-19. Seven studies [14, 15, 23, 24, 27, 30, 31] have shown that city lockdown, stay at home orders, traffic suspension and restrictions of mass gathering are strongly associated with reduced growth rate of COVID-19 confirmed cases and reduction in the epidemic growth. Moreover in their study Salvatore M et al. [23] found that lockdown was partly effective due to state level variations which should be considered in implementing lockdown. Adding to that Meo SA et al. [24] demonstrated that lockdown alone will not be effective unless it is implemented with other interventions such as social distancing and community wide mask wearing and in their quasi experimental study Kepp KP et al. [31] suggested that efficient infection surveillance and voluntary compliance may make full lockdowns unnecessary at least in some circumstances. Six studies [14–16, 18, 25, 26] found that identification of cases with isolation, quarantine of close contacts adding to home quarantine have been effective in suppressing transmission of COVID-19. Social and physical distancing measures have been proven in eight of the included studies [14, 16, 17, 20–22, 28, 29] to decrease the transmission of COVID-19. Thu TPB et al. [20] showed in their study that the time of promulgating the social distancing measures partly influences the intervention outcomes, adding to that population densities, crowding and socio-economic variables as it was suggested by Krishnamachari B et al [29] Three studies [ 14, 19, 26] showed that compulsory mask wearing and community wide masking may contribute to the control of COVID-19 when they are implemented with other non-pharmaceutical control measures. In addition to that three studies [16, 18, 19] demonstrated that testing in conjunction with active case finding and contact tracing especially when implemented with isolation of cases and close contacts and social distancing are effective in reducing the transmission of COVID-19 and particularly important in maintaining suppression. Two studies [ 16, 17] suggested that school closures together with the restrictions of mass gathering and physical distancing measures may have an effect in reducing the transmission of SARS-COV-2.Pan A et al [ 14], found in their study conducted in Wuhan, China that the rate of cases among health workers was substantially higher than in the general population in the period with there is no strong public health interventions which indicated a high risk of nosocomial infections and which might be inadequate use of personal protective equipment and lower awareness. However after increasing awareness and wider use of personal protective equipment adding to hospital-level prevention and management in parallel with the implementation of strong public heath interventions the rate of confirmed cases quickly decreased and furthermore no new case were reported among local health workers which prove that protecting heath care workers is an important measure in controlling an outbreak of a high transmissible infectious disease. Finally Zeng K et al. [26] and Seong H et al. [21] suggested that early community mask wearing and timely border control interventions using modern digital tools in addition to early and timely measures with strengthened social distancing interventions should be implemented to suppress and control the COVID-19 pandemic effectively.

**Table 1:** Characteristics of included studies and main outcomes

Authors, country	Study design	Objective	Methods	Main outcomes
**Pan A et al** [14]**, China**	Interrupted time series	Evaluate the association of several public health interventions on the control of COVID 19 outbreak over 5 periods according to key interventions.	32, 583 laboratory-confirmed COVID-19 cases. Time periods and Interventions: 1st period: time before January 10, 2020 without specific intervention. 2nd period: January 10 to 22, 2020, no strong intervention, massive migration, first human-to-human transmission on January 20 and hospitals started to be crowded.3rd period: between January 23 and February 1, 2020 city lockdown, traffic suspension, home quarantine, social distancing measures including compulsory mask wearing in public places and cancellation of social gatherings.4th period: February 2 to 16, 2020 intensified measures with centralized quarantine and treatment, improved medical resources and stay at home policy.5th period: February 17th to March 8th 2020, centralized quarantine and community universal survey. Rt: effective reproduction number is the mean number of secondary cases generated by typical case at primary case is an indicator that measures SARS-CoV-2 before and after the intervention.	The daily confirmed case rate per million people increased from 2.0 (95% CI, 1.8–2.1) before January 10, to 45.9 (95% CI, 44.6–47.1) in the 2nd period, to 162.6 (95% CI, 159.9–165.3) in the 3rd period and then decreased to 77.9 (95% CI, 76.3–79.4) in the 4th period. After February 16, it decreased to 17.2 (95% CI, 16.6–17.8). Rt varied in the first period, gradually increased in the 2nd period with a peak of 3.82 on January 24, and then declined. It fell below 1.0 on February 6, 2020, and further decreased to below 0.3 on March 1, 2020.
**Wang K-W et** **al** [15]**, China**	Interrupted time series	Estimate the effects of wartime control measures after early imported COVID-19 cases in Jiangsu from Hubei Province.	Time series observations from January 2 2 to February 18, 2020. Wartime control measures: Collect estimate, report and release emergency information every day. Put cities with epidemic on lockdown to limit population mobility. Restrict or stop crowd gathering. Migrant management such as on-site isolation for confirmed COVID-19 cases and contact tracing. Traffic health quarantine.	From January 22to February 18, 2020 the number of confirmed cases increased from 1 to 631. No new confirmed cases were identified after February 18th.
**Cowling BJ et al** [16]**, Hong Kong**	Cohort Study	Quantify behavioural changes in population of Hong Kong during the COVID-19 outbreak and describe effect of public health interventions on COVID-19 and influenza transmission.	Public health interventions: Travel and border entry restrictions and bans, testing, tracing, flexible working arrangements, school closures, quarantine and isolation orders that has been issued for cases and their contacts and travellers arriving from affected countries, cancellation of many conferences, some religious organizations and local mass gatherings and social distancing. Rt: effective reproduction number, mean number of secondary infections that result from a primary case of infection at time t.	Public health interventions and population behaviour changes such social distancing and personal protective measures implemented in Hong Kong since January 2020 is associated with reduced spread of COVID-19. Contact tracing, quarantine and social distancing played an important role in suppressing transmission adding to case identification with isolation.
**Jüni P et al** [17]**, 144 geopolitical areas**	Cohort Study	Determine whether epidemic growth is associated with climate or public health interventions.	Prospective cohort study of 144 geopolitical areas with at least 10 cases and local transmission excluding China, South Korea, Iran and Italy. Determination of the association between epidemic growth and latitude, temperature, humidity, school closures, restrictions of mass gatherings and measures of social distancing during an exposure period from March 7 to 13, 2020) using weighted random-effects regression.	Few or no associations of epidemic growth with latitude and temperature, weak negative association with relative and absolute humidity. Strong associations for implemented public health interventions.
**Wang J et al** [18]**, China**	Longitudinal Study	Estimate the incidence of 2019nCoV infection among people who are under home quarantine in Shenzhen province, China.	Stratified multistage random sampling method has been used to recruit participants and collect demographic information and laboratory results of people under home quarantine. Descriptive analysis was conducted to estimate the basic characteristics and to calculate the Incidence of novel coronavirus (2019-nCoV) infection among people under home quarantine. In order to report the outcomes of categorical variables proportions and frequencies were used. Mean and range were used to express continuous variables.	Testing for a total of 2004 people was conducted and three of these tested positive for 2019nCoV.The incidence of COVID-19 in the sample was 1.5‰ (95% CI: 0.31‰–4.37‰). None of the three patients had obvious symptoms during the time of home quarantine. Also, they did not report any history of contacts with confirmed cases. Home quarantine has been effective in preventing the early transmission of COVID-19.
**Cheng VC-C et al** [19]**, China**	Cohort Study	Assess the effect of community-wide mask usage to control COVID-19 in Hong Kong Special Administrative Region (HKSAR). Analyze the incidence of COVID19 in geographical areas with or without community-wide masking.	During the first 100 days epidemiological analysis was performed for confirmed cases especially the ones that acquired COVID-19 during mask-off and mask-on settings. The incidence of COVID-19 per million populations in HKSAR with community-wide masking was compared to that of non-mask- wearing countries which are comparable with HKSAR in terms of population density, healthcare system, BCG vaccination and social distancing measures but not community-wide masking.	Epidemiological measures by the HKSAR government: border controls from day 36, followed by imposing home quarantine order for 14 days to all entrees from mainland China from day 40. Then, the quarantine order was progressively imposed to all entrees into HKSAR from day 80. All entrees were compulsorily tested for SARS-CoV-2 from day 100. In addition to isolation of confirmed cases, contact tracing and quarantine, closure of affected or high risk premises, and social distancing measures such as home-office and school closure were instituted. 961 cases of COVID-19 were confirmed in HKSAR on day 100. From day 31 to 71 there were 111 cases predominantly local cases and from day 72 there were 840 cases predominantly imported cases with local clusters of cases. Among the 961 confirmed cases, there were 11 clusters of 113 persons that were directly engaged in mask-off activities. There were only three clusters involving 11 persons engaged in mask-on settings at the workplace there were significantly more COVID-19 clusters involving mask-off settings.
**Thu TPB et al** [20]**, The U.S., Spain, Italy, The U. K, France, Germany, Russia, Turkey, Iran and China.**	Longitudinal Study	Present the effect of social distancing interventions on the spread of COVID-19 in the cases of 10 highly infected countries.	The relationships between the social distancing interventions and the statistics of COVID-19 confirmed-cases and deaths were analyzed in order to elucidate the effectiveness of the social distancing interventions on the spread of COVID-19 in 10 highly infected countries including The U.S., Spain, Italy, The U.K., France, Germany, Russia, Turkey, Iran and China.	It took between 1 to 4 weeks since the point of highest level of social distancing measures promulgation until the numbers of daily confirmed-cases and daily deaths showed signs of decreasing. The effectiveness of the social distancing measures on the spread of COVID-19 was different between the 10 studied countries. This variation is due to the difference in the level of promulgated social distancing measures and in the difference in the COVID-19 spread situation at the time of promulgation in these countries. The growth rate of daily confirmed-cases at the time of promulgating the social distancing measures partly influences the decline rates of daily confirmed-cases after the spread reached its peak.
**Seong H et al** [21]**, South Korea**	Cohort Study	Compare the epidemiologic features of the second and third waves of the coronavirus disease 2019 (COVID-19) pandemic in South Korea.	COVID-19 data were collected between 6 May and 30 December 2020. The degree of social activity was estimated using an Internet search trend analysis program for leisure-related keywords, including ‘eating-out’, ‘trip’ and ‘get directions’ (transportation). Demographics, transmission chains, case fatality rates, social activity levels and public health responses were compared between the second (13 August–18 September 2020) and third (4 November 2020–present) waves.	The 3rd wave was characterized by delayed strengthening of social distancing measures (3 vs.15 days), longer duration (36 vs. > 56 days) and a higher case fatality rate (0.91% vs.1.26%) compared to the 2nd WAVE. There were significant differences in transmission chains between the two waves (*P* < 0.01). In comparison with the second wave, the proportion of local clusters (24.8% vs. 45.7%) was lower in the third wave, and personal contact transmission (38.5% vs. 25.9%) and unknown routes of transmission (23.5% vs. 20.8%) were higher in the third wave. In conclusion early and timely interventions with strengthened social distancing policies should be implemented to suppress and control the COVID-19 pandemic effectively.
**Lam HY et al** [22]**, Hong Kong**	Longitudinal Study	Review the epidemiology of the confirmed COVID-19 cases reported between January to May 2020 Assess the overall effectiveness of the various public health Interventions.	Description and comparison of the epidemiological and clinical characteristics of the cases recorded in different phases of the epidemic. Using the changes in the daily number of confirmed cases and the interval from symptom onset to hospital admission the effectiveness of the public health interventions implemented were reviewed.	Several public health interventions such as enhanced surveillance, border control, and social distancing, were introduced in phases in response to the rapid spread of the coronavirus locally and globally. Overall, the combination of public health interventions taken in Hong Kong were associated with a stabilization of case numbers and absence of a community-wide COVID-19 outbreak during the 4.5 m following the reporting of the first case.
**Salvatore M et al** [23]**, India**	Longitudinal Study	Evaluate the effect of four-phase national lockdown from March 25 to May 31 in response to the COVID-19 pandemic in India.	Participants Confirmed COVID-19 cases nationally and across 20 states that accounted for > 99% of the current cumulative case counts in India until 31 May 2020. Exposure Lockdown (non-medical intervention).	Results The estimated effective reproduction number R for India was 3.36 (95% CI 3.03 to 3.71) on 24 March, whereas the average of estimates from 25 May to 31 May stands at 1.27 (95% CI 1.26 to 1.28). Patterns of change over lockdown periods indicate the lockdown has been partly effective in slowing the spread of the virus at the national level. However, there exist large state-level variations and identifying these variations can help in both understanding the dynamics of the pandemic and formulating effective public health interventions.
**Meo SA et al** [24]**, 27 countries**	Cohort Study	Assess the impact of 15 days before, 15 days during, and 15 days after the lockdown on the the prevalence and mortality rate in 27 countries during COVID-19 pandemic.	27 countries were randomly selected and the information on the trends in the prevalence and mortality due to COVID-19 pandemic in was taken from World Health. Organization and lockdown data were obtained from studied countries and their ministries. Analysis of the impact of lockdown for 15 days before, 15 days during, and 15 days after the lockdown on the prevalence and mortality due to the COVID-19 pandemic in 27 countries.	Daily cases of SARS-COV-2 and the growth factor results declined and the growth rate per day both declined to an impressive negative level in the case of the growth rate per day by the time period of 15 days after the lockdown period, these two metrics of infection spreading did not fall sufficiently to control the pandemic. Lockdown policies should adhere to optimizing behaviour such as social distancing measures and community wide mask wearing that can affect spreading the COVID-19 pandemic. Lockdown alone will not be effective.
**Xu T-L et al** [25]**, China**	Longitudinal Study	Summarize the containment measures taken in China, as well as the effect of the practices on SARS-CoV-2 transmission.	The measures taken by the governments was tracked and sorted on a daily basis from the websites of governmental authorities. The measures were reviewed and summarized by categorizations, figures and tables. The population shift levels, daily local new diagnosed cases, daily mortality and daily local new cured cases were used for measuring the effect of the interventions.	Practices were categorized into active case surveillance, rapid case diagnosis and management, strict follow-up and quarantine of persons with close contacts. Together with these measures, daily local new diagnosed cases, and mortality rates were decreased and the daily local new cured cases were increased in China. China’s practices are effective in controlling transmission of SARS-CoV-2.
**Zeng K et al** [26]**, United States, Spain, and Italy, with Taiwan, South Korea, and Singapore**	Longitudinal study	Compare the measures taken against the spread of COVID-19 in the United States, Spain, and Italy, with Taiwan, South Korea, and Singapore, especially related to the use of digital tools for contact tracing.	COVID-19 death rate information were taken from the European Centre for Disease Prevention and Control (ECDC), accessed through the Our World in Data database and were evaluated based on population size per 100,000 people from December 31, 2019, to September 6, 2020. All policies and interventions were obtained from their respective governmental websites.	Strong association between lower death rates per capita and countries that implemented early mask use and strict border control measures that included mandatory quarantine using digital tools. There was a significant difference in the number of deaths per 100,000 when comparing Taiwan, South Korea, and Singapore with the United States, Spain, and Italy. These findings suggest that early intervention with the use of digital tools had a strong correlation with the successful containment of COVID-19. Infection rates and subsequent deaths in Italy, Spain, and the United States could have been much lower with early community mask wearing and more importantly timely border control interventions using modern digital tools.
**Wong CKH et al** [27]**, 54 countries and 4 epicentres of the COVID-19 pandemic (Wuhan, New York State, Lombardy, and Madrid),**	Longitudinal study	Describe and evaluate the impact of national containment interventions and policies such as stay-at-home orders, curfews, and lockdowns on decelerating the increase in daily new cases of COVID-19 rates in 54 countries and 4 epicentres of the pandemic worldwide.	The effective dates of the national containment interventions were reviewed of 54 countries and 4 epicenters of the COVID-19 pandemic (Wuhan, New York State, Lombardy, and Madrid) and cumulative numbers of confirmed COVID-19 cases and daily new cases provided by health authorities were searched. Data were drawn from an open, crowdsourced, daily-updated COVID-19 data set provided by Our World in Data. Moreover the trends in the percent increase in daily new cases from 7 days before to 30 days after the dates on which containment measures went into effect by continent, World Bank income classification, type of containment interventions, effective date of containment interventions and number of confirmed cases on the effective date of the containment measures were examined as well.	122,366 patients with confirmed COVID-19 infection from 54 countries and 24,071 patients from 4 epicentres on the effective dates on which stay-at-home orders, curfews, or lockdowns were implemented from January 23 to April 11, 2020 were included in this study. Stay-at-home, curfew, and lockdown interventions commonly started in countries with approximately 30, 20%, or 10% increases in daily new cases. All three interventions were found to lower the percent increase in daily new cases to < 5 within one month. 20% had an average percent increase in daily new cases of 30–49 over the seven days prior to the implementation of the containment measures; the percent increase in daily new cases in these countries was curbed to 10 and 5 a maximum of 15 days and 23 days after the implementation of containment interventions, respectively. Different national containment interventions were associated with a decrease in daily new cases of confirmed COVID-19 infection. Stay-at-home orders, curfews, and lockdowns curbed the percent increase in daily new cases to < 5 within a month.
**Siedner MJ et al** [28]**, USA**	Longitudinal study	Estimate the change in COVID-19 case growth before and after implementation of statewide social distancing measures in the US.	The primary exposure was time before (14 days prior to and through 3 days after) versus after (beginning 4 days after, to up to 21 days after) implementation of the first state-wide social distancing interventions. State-wide restrictions on internal movement were examined as a secondary exposure. The COVID-19 case growth rate was the primary outcome. The COVID-19-attributed mortality growth rate was the secondary outcome.	Statewide social distancing interventions were associated with a decrease in the COVID-19 case growth rate that was statistically significant. Statewide social distancing interventions were also associated with a decrease in the COVID-19-attributed mortality growth rate beginning 7 days after implementation; however this decrease was no longer statistically significant by 10 days.
**Krishnamachari B et al** [29]**, USA (preprint)**	Cohort Study	Examine the effects of government implemented social distancing measures on the cumulative incidence rates of COVID-19 in the United States on a state level and in the 25 most populated cities	Assessed social distancing variables: days to closing of non-essential business; days to stay home orders; days to restrictions on gathering, days to restaurant closings and days to school closing. Using negative binomial regression, adjusted rate ratios and 95% confidence intervals were calculated in order to compare two levels of a binary variable: “above median value,” and “median value and below” for days to implementing a social distancing interventions. For city level data, the effects of these social distancing variables were assessed as well in high (above median value) vs low (median value and below) population density cities. For the state level analysis, days to school closing was associated with cumulative incidence, with an adjusted rate ratio of 1.59 (95% CI:1.03,2.44), *p* = 0.04 at 35 days.	The effect of social distancing interventions may differ between states and cities and between locations with different population densities. Individual approaches are needed to containment of an epidemic, with an awareness of their own structure in terms of crowding and socio-economic variables.
**Singh BB et al** [30]**, India (preprint)**	Longitudinal study	Evaluation of the public health interventions using the effective reproduction number (Rt), in key lockdown periods in India.	Laboratory-confirmed COVID-19 infections rates per day and effective reproduction number (Rt) were estimated for 4 periods (Pre-lockdown and Lockdown Phases 1 to 3) according to nationally implemented phased interventions. Adoption of these measures was estimated using Google mobility data. Estimates at the national level and for 12 Indian states most affected by COVID-19 are presented. Using data are publicly available from Google a domain-specific mobility index was constructed using India’s mobility report (Google Inc., Mountain View, CA, USA). domain-specific mobility index was constructed for the country and 12 Indian states.	Median mobility in India decreased in all contact domains, with the lowest being 21% in retail/recreation (95% CI 13–46%), except home which increased to 129% (95% CI 117–132%) compared to the 100% baseline value. The Indian government imposed strict contact mitigation, followed by a phased relaxation, which slowed the spread of COVID-19 epidemic progression in India.
**Kepp KP et al** [31]**, Denmark (preprint)**	Quasi experimental study	Analyse the unique case-controlled epidemiological dataset arising from the selective lockdown of parts of Northern Denmark, but not others, as a consequence of the spread of mink-related mutations in November 2020.	A quasi-natural experiment in the Danish region of Northern Jutland. 7 of the 11 municipalities of the region went into extreme lockdown in early November after the discovery of mutations of Sars-CoV-2 while the four other municipalities retained the moderate restrictions of the remaining country. Incidentally, the infection numbers in the two groups were compared.	While infection levels decreased, they did so before lockdown was effective. Infection numbers decreased as well in other municipalities without mandates. Control of infection pockets possibly together with voluntary social behaviour was apparently effective before the mandate which explains why the infection decline occurred before and in both the mandated and non-mandated areas. The findings of this study suggest that efficient infection surveillance and voluntary compliance make full lockdowns unnecessary at least in some circumstances.

**Table 2:** Quality assessment of the included studies

Article	Selection bias	Study design	Confounders	Blinding	Data collection methods	Withdrawals and drop-outs	Global rating
**Pan A et al** [14]	Moderate	Moderate	N/A	N/A	weak	N/A	Moderate
**Wang K-W et al** [15]	Moderate	Moderate	N/A	N/A	weak	N/A	Moderate
**Cowling BJ et al** [16]	Moderate	Moderate	weak	N/A	Moderate	N/A	Moderate
**Jüni P et al** [17]	Moderate	Moderate	Weak	N/A	weak	N/A	weak
**Wang J et al** [18]	Strong	Moderate	N/A	N/A	Moderate	Strong	Strong
**Cheng VC-C et al** [19]	Moderate	Moderate	Weak	N/A	Moderate	N/A	Moderate
**Thu TPB et al** [20]	Moderate	Moderate	N/A	N/A	Moderate	N/A	Moderate
**Seong H et al** [21]**,**	Moderate	Moderate	Weak	N/A	Moderate	N/A	Moderate
**Lam HY et al** [22]	Moderate	Moderate	N/A	N/A	Moderate	N/A	Strong
**Salvatore M et al** [23]	Moderate	Moderate	N/A	N/A	Weak	N/A	Moderate
**Meo SA et al** [24]	Moderate	Moderate	Weak	N/A	Moderate	N/A	Moderate
**Xu T-L et al** [25]	Moderate	Moderate	N/A	N/A	Moderate	N/A	Moderate
**Zeng K et al** [26]	Moderate	Moderate	N/A	N/A	Weak	N/A	Moderate
**Wong CKH et al** [27]	Moderate	Moderate	N/A	N/A	Weak	N/A	Moderate
**Siedner MJ et al** [28]	Moderate	Moderate	N/A	N/A	Weak	N/A	Moderate
**Krishnamachari B et al** [29]	Moderate	Moderate	Moderate	N/A	Weak	Moderate	Moderate
**Singh BB et al** [30]	Weak	Moderate	N/A	N/A	Weak	N/A	Weak
**Kepp KP et al** [31]	Moderate	Strong	Strong	Weak	Moderate	N/A	Moderate

**Table 3:** Level of evidence of the included studies and grade of recommendation

***Article***	***Type of study***	***Level of evidence***	***Grade of recommendation***
**Pan A et al** [14]	Interrupted time series	2+	C
**Wang K-W et al** [15]	Interrupted time series	2+	C
**Cowling BJ et al** [16]	Cohort Study	2-	C
**Jüni P et al** [17]	Cohort Study	2-	C
**Wang J et al** [18]	Longitudinal Study	2++	B
**Cheng VC-C et al** [19]	Cohort Study	2-	C
**Thu TPB et al** [20]	Longitudinal Study	2+	C
**Seong H et al** [21]**,**	Cohort Study	2-	C
**Lam HY et al** [22]	Longitudinal Study	2+	C
**Salvatore M et al** [23]	Longitudinal Study	2+	C
**Meo SA et al** [24]	Cohort Study	2-	C
**Xu T-L et al** [25]	Longitudinal Study	2+	C
**Zeng K et al** [26]	Longitudinal study	2+	C
**Wong CKH et al** [27]	Longitudinal study	2+	C
**Siedner MJ et al** [28]	Longitudinal study	2+	C
**Krishnamachari B et al** [29]	Cohort Study	2+	C
**Singh BB et al** [30]	Longitudinal study	2-	C
**Kepp KP et al** [31]	Quasi experimental study	1+	A

## Discussion

We found that public health interventions and non-pharmaceutical control measures were effective in reducing the transmission of COVID-19 and were associated with reduced epidemic growth. The identified studies showed that travel restrictions, borders measures, quarantine of travellers arriving from affected countries, city lockdown, restrictions of mass gathering, isolation and quarantine of confirmed cases and close contacts, social distancing measures, compulsory mask wearing, contact tracing and testing, school closures and personal protective equipment use among health workers were effective in mitigating the spread of COVID-19 with varying degrees. Our results are in line with the findings of other studies [32–34] that demonstrated that public health measures and non-pharmaceutical control strategies are effective in mitigating the current pandemic of COVID-19 and in some countries aggressive and extreme interventions are probably needed to bring the epidemic under control and to prevent very large number of deaths and excess hospitals capacities.

Travel and entry restrictions, borders measures and quarantine of travellers arriving from affected countries were effective in controlling the spread of infection caused by SARS-CoV 2. Those interventions have been shown to be effective as well in other studies [ 6, 35], which suggest that travel restrictions and border control measures including surveillance targeting inbound travellers from affected countries and 14-day quarantine for arriving passengers adding to other public health interventions were associated with a stabilization of case numbers.

City lockdown, restriction of mass gathering physical distancing and stay at home policies has been shown to be effective as well in reducing the spread of SARS-CoV2 in the current study. Further studies support these findings and showed that lockdown measurements and stay at home orders were efficient in controlling and slowing down the spread of the epidemic [36–39] were strongly associated with the containment of COVID-19 [40]. A rapid review of modelling studies [ 41] found that quarantine is crucial in decreasing incidence and mortality in the pandemic of COVID-19. Moreover in order to ensure effectiveness it is very important implement quarantine measures especially in combination with other public health interventions at the early stage of the epidemic. Adding to that, in their study Marco Vinceti et al [ 42] showed the less rigid lockdown measurements led to an insufficient reduction in transmission to reverse the outbreak and with a tighter lockdown mobility and person to person transmission decreased enough to bring down transmission straight off below the level required to counteract spread of SARS-CoV-2 infection. In addition to that physical distancing strategies and restriction of human mobility [ 43, 44] has been showed to have a notable effect on controlling the spread of the COVID-19 outbreak.

Isolation and quarantine measures of contacts and close contacts adding to contact tracing are crucial to control the outbreak of COVID-19 and reduce the human to human transmission. Those results are consistent with the findings of other studies [45–48] which indicate successful contact tracing and isolation of cases and close contacts are highly important to control the outbreak and to ensure a lower reproduction number below 1. These interventions might be more effective if combined with other measures such as physical distancing, self-isolation and testing. Testing is a key intervention in mitigating the spread of COVID-19 especially when it is applied in conjunction with tracing and isolation of cases and close contacts [49].

Compulsory mask wearing and community wide masking policies are essential in controlling the pandemic of COVID-19. Authors of a rapid systematic review [50] on the efficacy of face masks suggest that masking wearing could be beneficial in the context of COVID-19 outbreak especially universal community mask use and in the health care settings as well. Findings from a systematic review and meta-analysis [51] showed that mask wearing by health workers and non-health workers and in the general community is and efficient in preventing the infection by SARS-CoV2. Another study [52] showed that wearing masks in public is crucial as a preventive measure to ensure a significant reduction in the daily infected cases. In addition to that a prospective cohort study [53] found that the risk of infection by SARS-CoV-2 is increase among frontline health workers, therefore adequate strategies should be implemented to ensure the availability of personal protective equipments in order to protect health workers from COVID-19. Moreover timing is very important while implementing non-pharmaceutical interventions which should be initiated early when the numbers of COVID-19 cases are low as it was demonstrated in an observational study conducted by Qureshi A I et al [54]

School closures had been found to be effective in reducing the transmission of COVID-19, recently this intervention has been widely discussed; some studies [55, 56] found that school closures were associated with a reduction in the transmission of COVID-19 and in the mortality rate as well. However, other studies [57, 58] showed that school closures don’t have any mitigating impact on the transmission of COVID-19 as children are likely to be asymptomatic and they don’t seem to be greater transmitters in comparison with adults.

Further studies [59, 60] found additional tools that help prevent and control the COVID-19 pandemic such as internet hospitals and virtual care which presents a promising potential in the control of the COVID-19 outbreak as they are capable of reducing the emergency room visits, reducing the risk of nosocomial cross-infection by treating patients remotely, prevent the shortage of health care resources and promote personal prevention measures such as social distancing, mask wearing and hand hygiene. A systematic review [61] showed that telehealth is capable of minimizing the risk of COVID-19 transmission by decreasing the physical contacts adding to providing continuous community care.

This systematic review has several limitations, the included studies have heterogeneous methodology and most of them lack a control group and a vigorous study design. Although most of the included studies have moderate quality and for the remaining studies; two studies have low quality and only two studies have strong quality. Also, most of the included studies have moderate level of evidence and low grade of recommendation, six studies have low level of evidence and low grade of recommendation, only one study has moderate level of evidence and grade of recommendation and only one study as well has high level of evidence and grade of recommendation. In addition to that most of the public health interventions are implemented simultaneously or within a short period of time which means that it is difficult to evaluate the effect of each intervention alone accurately, consequently we can either underestimate or overestimate their impact on the COVID-19 pandemic. Future research studies which have rigorous methodology especially experimental and quasi experimental studies are needed to properly evaluate the outcomes of these public health interventions and non-pharmaceutical measures.

## Conclusion

With no effective treatment and vaccine against SARS-CoV-2, public health measures and non-pharmaceutical interventions are vital to reduce the infection and mortality rate. Some interventions are not efficient enough when implemented alone and could not contain the outbreak, thus, depending on the country and the phase of the epidemic multiple interventions are needed to be applied together in order to bring the outbreak under control.

## Data Availability

Protocol of this systematic review could be found at https://www.crd.york.ac.uk/prospero/display_record.php?RecordID=196018
